# EpsA is an essential gene in exopolysaccharide production in *L* *actobacillus johnsonii* FI9785

**DOI:** 10.1111/1751-7915.12314

**Published:** 2015-09-24

**Authors:** Enes Dertli, Melinda J. Mayer, Ian J. Colquhoun, Arjan Narbad

**Affiliations:** ^1^ Department of Gut Health and Food SafetyInstitute of Food ResearchNorwichColneyNR4 7UAUK; ^2^ Analytical Sciences UnitInstitute of Food ResearchNorwichColneyNR4 7UAUK; ^3^ Department of Food EngineeringFaculty of EngineeringBayburt UniversityBayburt69000Turkey

## Abstract

*L* *actobacillus johnsonii* FI9785 has an eps gene cluster which is required for the biosynthesis of homopolymeric exopolysaccharides (EPS)‐1 and heteropolymeric EPS‐2 as a capsular layer. The first gene of the cluster, epsA, is the putative transcriptional regulator. In this study we showed the crucial role of epsA in EPS biosynthesis by demonstrating that deletion of epsA resulted in complete loss of both EPS‐1 and EPS‐2 on the cell surface. Plasmid complementation of the epsA gene fully restored EPS production, as confirmed by transmission electron microscopy and nuclear magnetic resonance (NMR) analysis. Furthermore, this complementation resulted in a twofold increase in the expression levels of this gene, which almost doubled amounts of EPS production in comparison with the wild‐type strain. Analysis of EPS by NMR showed an increased ratio of the heteropolysaccharide to homopolysaccharide in the complemented strain and allowed identification of the acetylated residue in EPS‐2 as the (1,4)‐linked βGlc*p* unit, with the acetyl group located at *O*‐6. These findings indicate that epsA is a positive regulator of EPS production and that EPS production can be manipulated by altering its expression.

## Introduction

Exopolysaccharides (EPS) are one of the key components of the cell surface of lactic acid bacteria (LAB) and have a considerable impact on their surface characteristics (Broadbent *et al*., 2003). EPS can be either bound to the cell surface or secreted to the environment; structurally, they are classified as homopolysaccharides, which are composed of one type of sugar monomer, and heteropolysaccharides, which contain two or more sugar subunits and sometimes other organic molecules in their structure (Welman and Maddox, 2003; Badel *et al*., 2011). A single gene defined as *gtf* is commonly responsible for the production of homopolymeric glucose EPS in LAB (Walter *et al*., 2008; Badel *et al*., 2011). The biosynthesis of heteropolymeric EPS is more complex and a cluster of 8–17 genes is required, including conserved genes for regulation, repeat unit biosynthesis, chain length determination, polymerization and export (Cieslewicz *et al*., 2001; Jolly and Stingele, 2001; Broadbent *et al*., 2003; Peant *et al*., 2005; Berger *et al*., 2007; Lebeer *et al*., 2009). EPS produced by LAB have a great variety of structures and have many applications in the dairy industry, particularly in improving texture and as thickening and stabilizing agents (Welman and Maddox, 2003; Badel *et al*., 2011). Consequently the production of novel or modified structures and strategies to increase production in LAB are of significant industrial interest.

In addition to their utility in industry, EPS produced by LAB have several biological applications, showing immunomodulatory (Sims *et al*., 2011; Fanning *et al*., 2012), anti‐tumour (Kitazawa *et al*., 1998) and cholesterol‐lowering activities (Nakajima *et al*., 1992). EPS can also be involved in some important processes related to probiotic properties including biofilm formation (Lebeer *et al*., 2008; Vu *et al*., 2009), auto‐aggregation (Walter *et al*., 2008; Dertli *et al*., 2015), colonization (Denou *et al*., 2008; Walter *et al*., 2008; Fanning *et al*., 2012) and survival (Mozzi *et al*., 2009; Fanning *et al*., 2012). Thus, understanding how to manipulate EPS production can also have importance in the biological performance of probiotics.

*Lactobacillus johnsonii* FI9785 has been shown to act as a competitive exclusion agent against *Clostridium perfringens* and other pathogens in poultry (La Ragione *et al*., 2004). The mechanism of the exclusion process is still undefined, but one of the proposed modes of action is that the bacteria adhere to the gastrointestinal tract and prevent the colonization of pathogenic bacteria (Reid and Burton, 2002), a process which is related to cell surface characteristics (Lebeer *et al*., 2008). Recently, we demonstrated that *L. johnsonii* FI9785 produces two different EPS: homopolymer EPS‐1 is a branched dextran with every backbone residue substituted with a 2‐linked glucose unit, and heteropolymer EPS‐2 has a hexasaccharide repeating unit composed of two galactose and four glucose residues with different types of linkages between each sugar residue (Dertli *et al*., 2013). The genome of *L. johnsonii* FI9785 does not contain a classical *gtf* gene. However, an *eps* gene cluster with a similar organizational structure to those associated with heteropolymeric EPS production was identified, with 14 putative genes related to EPS biosynthesis (Horn *et al*., 2013). Nuclear magnetic resonance (NMR) analysis of a mutant where the *eps* cluster had been deleted showed that this cluster was required for the biosynthesis of both EPS‐2 and EPS‐1 (Dertli *et al*., 2013). Mutations in this cluster resulted in alterations in phenotype, EPS production, surface characteristics and cell–cell interactions (Horn *et al*., 2013; Dertli *et al*., 2015). The first gene in the cluster, *epsA*, has homology to the LytR family of transcriptional regulators; in *Streptococcus pneumoniae* deletion of a similar gene *cpsIaA* caused a reduction in capsule production (Cieslewicz *et al*., 2001).

In the current study, we explored the importance of the *epsA* gene by demonstrating that deletion of *epsA* resulted in an acapsular phenotype, while complementation by plasmid expression fully restored EPS production and resulted in an almost twofold increase in EPS compared with the wild type. This demonstrates that *epsA* controls EPS biosynthesis and can be used to either prevent or increase its production.

## Results and discussion

**Figure 1:**
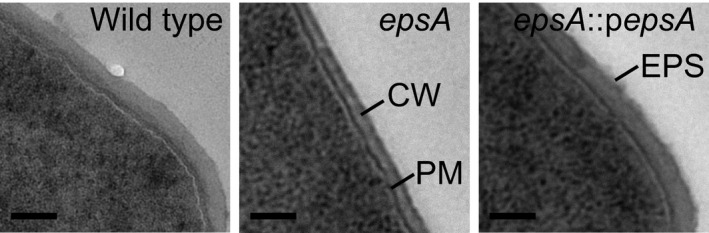
Transmission electron microscopy of wild type and mutant strains. *L* *. johnsonii* FI9785 (wild type) and its derivatives ΔepsA, made by deletion mutagenesis using the thermosensitive pG^+^host9 vector system, and complemented strain ΔepsA::pepsA (see supporting information) were grown and visualized as described previously (Dertli *et al*., 2013). The bar represents 100 nm. CW, cell wall, PM, plasma membrane.

**Figure 2:**
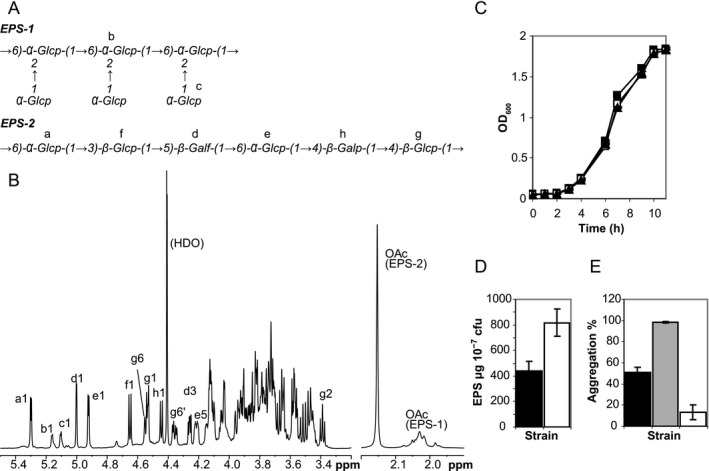
Analysis of EPS production and quality. A. Structure of the EPS‐1 and EPS‐2 produced by *L* *. johnsonii* FI9785 showing labelled sugar residues *b*, *c* from EPS‐1 and residues *a* and *d–h* from EPS‐2 (Dertli *et al*., 2013). B. 600 MHz ^1^H NMR spectrum (338°K, D_2_O) of EPS extracted from the ΔepsA::pepsA mutant. EPS were isolated from 500 ml cultures as previously described (Tallon *et al*., 2003); lyophilized samples were dissolved in D_2_O and 1D and 2D NMR spectra were obtained using the same pulse sequences as described previously (Dertli *et al*., 2013). C. Growth of strains to stationary phase. Optical density (OD600) readings represent the mean of triplicate samples +/− standard deviation. ■, wild type; □, ΔepsA; ▲, Δ*epsA*::p*epsA*. D. Increase in EPS production after complementation. EPS were quantified using the phenol‐sulfuric acid method (DuBois *et al*., 1956) with glucose as a standard and expressed as a quantity of EPS production per 10^7^ cfu (mean of triplicate samples +/− standard deviation). Black, wild type; white, ΔepsA::pepsA. E. Aggregation of wild type and mutant strains. The aggregation percentage was measured after overnight incubation (16 h) by flow cytometry (Dertli *et al*., 2015). Results are the mean of triplicates +/− standard deviation. Black, wild type; grey, ΔepsA; white, ΔepsA::pepsA.

To investigate the importance of *epsA*, the *epsA* coding sequence was knocked out by in‐frame deletion mutagenesis and this deletion strain (*ΔepsA*) was also complemented with the wild‐type *epsA* gene on a plasmid under the control of a constitutive promoter (Δ*epsA*::p*epsA*, see supporting information). There were no alterations in the growth rate or colony phenotype in comparison with the wild‐type strain (Fig. 2C); however, transmission electron microscopy analysis showed the absence of an EPS layer in Δ*epsA* while the cell walls of the wild type and Δ*epsA*::p*epsA* strains appeared thicker and less distinct due to the accumulation of EPS (Fig. 1). Cell surface‐associated EPS were isolated from cell pellets of both mutant strains and were subjected to NMR analysis to determine their structures (Fig. 2A and B). Analysis and quantification of EPS confirmed that *ΔepsA* was unable to produce either EPS‐1 or EPS‐2, resulting in an acapsular phenotype. Complementing the *epsA* gene in this mutant fully restored the biosynthesis of both EPS.

The ^1^H NMR spectrum of the Δ*epsA*::p*epsA* mutant (Fig. 2B) showed the presence of both EPS‐1 and EPS‐2. There was an increased ratio of EPS‐2 to EPS‐1, which contrasted with the composition of the mixture obtained from the wild type (also isolated from the bacterial cell pellet) in which EPS‐1 was the major polysaccharide (Dertli *et al*., 2013). ^1^H NMR spectra of the wild type (WT) and Δ*epsA* are provided for comparison with Δ*epsA*::p*epsA* as Supporting information (Fig. S1). Previously, acetyl groups were identified but not assigned to specific residues (Dertli *et al*., 2013). However it was established that the acetyl group in the hexasaccharide repeating unit of EPS‐2 gives rise to a sharp singlet at 2.15 ppm in the ^1^H spectrum (Fig. 2B); by integration there is one acetyl group per repeating unit. The other signals at ∼ 1.98 to 2.08 ppm are associated with EPS‐1 and although integration shows that there is approximately one acetyl group per disaccharide repeating unit, the presence of multiple peaks means that these groups must be distributed across several locations. The data reported previously (Dertli *et al*., 2013) referred to non‐acetylated EPS‐2, which was the predominant component in the sample analysed from the smooth mutant *epsC^D88N^*. EPS‐2 from Δ*epsA*::p*epsA* was 100% acetylated in addition to being the major polysaccharide, and this, together with comparison with the spectrum of *epsC^D88N^*, enabled a more accurate analysis of acetylation. A detailed study of the 2D NMR spectra showed that in this preparation EPS‐2 was acetylated at just one position: C6 of the (1,4)‐linked β‐Glc*p* residue, *g*. The ^1^H and ^13^C chemical shifts of EPS‐2 are listed in Table 1. The location of the acetyl group in acetylated EPS‐2 is revealed by the downfield displacement, compared with non‐acetylated EPS‐2, of the *g*6 signals to δ4.36/4.55 from δ3.84/4.00 (^1^H) and to δ65.73 from δ63.04 (^13^C). Shifts of neighbouring atoms (*h*1, *g*5) are also affected by the presence of the acetyl group as indicated in Table 1; the remaining shifts are essentially unchanged.

**Table 1:** ^1^ H and ^13^C chemical shifts of ΔepsA::pepsA repeating unit

Label^a^	Unit		Chemical shift (ppm)^b^						
			1	2	3	4	5	6	*O*Ac
*a*	(1,6)αGlc*p*→3	H	5.30	3.58	3.74	3.52	4.14	3.88, 4.11	
		C	101.94	74.43^c^	75.75^d^	72.27	73.80	71.43	
*f*	(1,3)βGlc*p*→5	H	4.65	3.45	3.65	3.65	3.47	3.74, 3.92	
		C	104.85	74.79	85.81	72.76	78.27	63.56	
*d*	(1,5)βGal*f*→6	H	5.00	4.13	4.26	4.11	4.05	3.82	
		C	110.41	83.63	79.02	84.42	80.60	63.99	
*e*	(1,6)αGlc*p*→4	H	4.92	3.57	3.74	3.48	4.22	3.72, 3.95	
		C	102.86	74.50^c^	75.53^d^	72.37	73.80	69.24	
*h*	(1,4)βGal*p*→4	H	**4.44**	3.57	3.72	4.03	3.79	3.84, 3.91	
		C	106.29	73.70	75.02	80.31	78.19	63.05	
*g*	(1,4)βGlc*p*→6	H	4.53	3.39	3.67	3.71	**3.80**	**4.36, 4.55**	2.15
		C	105.51	75.64^d^	77.06	81.94	**75.02**	**65.73**	23.05

**a.** The residues are listed in the order in which they occur in the linear repeating unit of the acetylated EPS‐2, i.e. *a* is linked to *f*, *f* to *d* etc. and *g* to *a*. The repeating unit is acetylated at C6 of residue *g*. **b.** Shifts which differ most from the previously reported (Dertli *et al*., 2013) non‐acetylated EPS‐2 are indicated in bold. **c,d.** ^13^C assignments with the same superscript letter may need to be interchanged.

The acetylation of bacterial EPS and plant polysaccharides has been shown to contribute to the gelling properties of these biopolymers (Robijn *et al*., 1995; Huang *et al*., 2002) and it was also reported that acetylation of the alginate produced by *Pseudomonas aeruginosa* increases the *in vivo* adherence of this pathogen to lung epithelium in cystic fibrosis patients (Riley *et al*., 2013). Although some functions have been attributed to the acetylation of the polysaccharides in both Gram‐positive and Gram‐negative bacteria, such as the provision of additional protection against the many types of hydrolases produced by gut bacteria, the acetylation mechanism of EPS has not been identified in detail. It was shown that an inner membrane protein WecH was responsible as an acetyltransferase for the *O*‐acetylation of the cell surface polysaccharide of *Escherichia coli* K12, and the gene encoding WecH was not located in the gene cluster responsible for the production of this polysaccharide (Kajimura *et al*., 2006). More recently a gene within the *algD* operon of the Gram‐negative *P. aeruginosa* responsible for alginate production, designated as *Algx*, was shown to be an acetyltransferase responsible for the acetylation of alginate (Riley *et al*., 2013). From Gram‐positive bacteria, a gene required for the *O*‐acetylation of EPS from *Staphylococcus aureus* (*cap5h*) was identified and shown to have a positive effect on colonization of mice (Bhasin *et al*., 1998), and *epsH* from the *eps* gene cluster of *Streptococcus thermophilus* Sfi6 showed homology to this gene (Stingele *et al*., 1999). None of the predicted gene products from the *L. johnsonii* FI9785 *eps* cluster show amino acid homology to acetyltransferases, but the genome harbours several putative acetyltransferase genes (Wegmann *et al*., 2009). Most of these are associated with the acetylation of core cell wall components, but a novel gene/s within this set could be responsible for the acetylation of EPS; the translated product of one locus, FI9785_RS04565, has homology to similar domains to those found in EpsH and Cap5H.

Having established that the *L. johnsonii* Δ*epsA*::p*epsA* strain was able to produce both EPS types, we subsequently investigated the EPS production levels in this mutant. Quantitative polymerase chain reaction (qPCR) analysis was performed to compare the *epsA* gene expression levels in wild type, Δ*epsA*::p*epsA* and Δ*epsA* mutant cells (see Supporting information). The expression of the *epsA* gene in the Δ*epsA* mutant could not be detected by qPCR analysis as expected (data not shown). In contrast, complementation of the *epsA* gene in the Δ*epsA*::p*epsA* mutant resulted in a 1.99 ± 0.34‐fold increase in the *epsA* gene expression level compared with that in the wild type. This was reflected in the accumulation of EPS – overexpression of *epsA* resulted in clearly increased levels of total EPS production, giving 817.6 +/− 108.4 μg/10^7^ colony‐forming units (cfu) compared with the wild type (441.2 +/− 73.5 μg/10^7^ cfu) (Fig. 2D).

Deletion of the putative transcriptional regulator of a similar heteropolysaccharide cluster in *S. pneumoniae* led to a reduction in capsule production, but not to a complete absence (Cieslewicz *et al*., 2001). Our experiments suggest a simple relationship between *epsA* gene expression and EPS production under these growth conditions, although expression analysis of the genes of the *eps* cluster is required to confirm the relationship between *epsA* and transcriptional regulation of EPS. In other studies, the relationship between transcription and production is not so direct: the EPS production of four *Lactobacillus rhamnosus* strains varied widely but the transcription levels of genes in *eps* clusters were similar (Peant *et al*., 2005), while EPS production in *Lactococcus lactis* strains varied with sugar source without changes in the activity of the *eps* promoter (Looijesteijn *et al*., 1999). Several factors can affect the levels of EPS production including the availability of sugar nucleotides (Looijesteijn *et al*., 1999; Boels *et al*., 2001; Levander *et al*., 2002), the expression level of genes in sugar catabolism pathways (Looijesteijn *et al*., 1999; Levander *et al*., 2002) and the carbon source utilized by the bacteria, as well as the transcriptional level of the genes responsible for EPS production (Looijesteijn *et al*., 1999; Audy *et al*., 2010). Previously, it was reported that a higher transcription level of the priming glycosyltransferase resulted in higher EPS production in *Bifidobacterium longum* subsp. *longum* CRC 002 (Audy *et al*., 2010). In another study the overexpression of the fructosebiphosphatase, which is a key enzyme in the biosynthesis of sugar nucleotides from fructose, resulted in increased EPS production in *L. lactis* (Looijesteijn *et al*., 1999). Similarly, it was shown that an almost threefold increase in the expression of an eps gene cluster in *L. lactis* gave a nearly fourfold increase in EPS production, although there was a reduction in the cell growth, potentially due to the fact that the sugar nucleotides are used not only in EPS production but also for bacterial metabolic activities including cell wall biosynthesis (Boels *et al*., 2003). In the current study the increased EPS production in *L. johnsonii* Δ*epsA*::*pepsA* did not significantly change the growth profile. It should be noted that although the *eps* cluster is required for the biosynthesis of both EPS, overexpression of *epsA* resulted particularly in an enhanced production of heteropolysaccharide EPS‐2, which supports our previous suggestion that a novel gene outside of the *eps* cluster might be involved in EPS‐1 production in conjunction with a gene/s in the *eps* cluster (Dertli *et al*., 2013).

The complete loss of an EPS layer in the Δ*epsA* mutant resulted in significantly higher aggregation levels (almost 100%) compared with the wild type (51.2 +/− 4.5%), while the increased EPS production in the Δ*epsA*::p*epsA* complemented strain resulted in a sharp decrease in aggregation to only 13.3 +/− 6.9% (Fig. 2E). These results agree with previous analysis of *L. johnsonii* mutants with reduced or increased EPS production, which found that a Δ*epsE* strain which only produced a thin layer of EPS‐1 also exhibited nearly 100% aggregation (Horn *et al*., 2013; Dertli *et al*., 2015); here, a similar level of aggregation with no EPS indicates that cell surface components revealed by the reduction in the EPS layer are more likely to be the positive agents of aggregation than EPS‐1.

Adhesion to HT29 cells, measured as described previously (Horn *et al*., 2013), was also increased in the acapsular Δ*epsA* mutant (18.4 +/− 0.3%) compared with the wild type (13.7 +/− 0.3%), while complementation in Δ*epsA*::p*epsA* restored the adhesion level to wild type levels (13.5 +/− 1.6%) despite the reduction in aggregation. Previously, increased EPS production in a smooth colony mutant *epsC^D88N^* produced a reduction in adhesion, and the finding here that a greater increase in EPS content still gave wild type adhesion levels lends further weight to suggestions that other factors such as chain length, attachment or ratio between EPS types have an impact on cell surface characteristics (Horn *et al*., 2013).

In conclusion, the current report shows that *epsA* is required for the biosynthesis of both homopolymeric EPS‐1 and heteropolymeric EPS‐2. The analysis of EPS production and *epsA* gene expression in the Δ*epsA*::*pepsA* mutant indicates that EPS production can be almost doubled by overexpressing the putative transcriptional regulator without any disruption in cell growth.

## Conflict of Interest

The authors confirm that there are no conflicts of interest.

## Supporting information


**Fig. S1.** 600 MHz ^1^H NMR spectra of EPS (300°K, D_2_O) isolated from WT, Δ*epsA* and Δ*epsA*::p*epsA* strains.
**Table S1.** Primers designed for *epsA*, 16S and *gyrB* genes for qPCR analysis.

## References

[mbt212314-bib-0001] Audy, J. , Labrie, S. , Roy, D. , and Lapointe, G. (2010) Sugar source modulates exopolysaccharide biosynthesis in *Bifidobacterium longum* subsp. *longum* CRC 002. Microbiol 156: 653–664. 10.1099/mic.0.033720-0 19850611

[mbt212314-bib-0002] Badel, S. , Bernardi, T. , and Michaud, P. (2011) New perspectives for Lactobacilli exopolysaccharides. Biotechnol Adv 29: 54–66. 20807563 10.1016/j.biotechadv.2010.08.011

[mbt212314-bib-0003] Berger, B. , Pridmore, R.D. , Barretto, C. , Delmas‐Julien, F. , Schreiber, K. , Arigoni, F. , and Brussow, H. (2007) Similarity and differences in the *Lactobacillus acidophilus* group identified by polyphasic analysis and comparative genomics. J Bacteriol 189: 1311–1321. 17142402 10.1128/JB.01393-06 PMC1797336

[mbt212314-bib-0004] Bhasin, N. , Albus, A. , Michon, F. , Livolsi, P.J. , Park, J.S. , and Lee, J.C. (1998) Identification of a gene essential for O‐acetylation of the *Staphylococcus aureus* type 5 capsular polysaccharide. Mol Microbiol 27: 9–21. 9466251 10.1046/j.1365-2958.1998.00646.x

[mbt212314-bib-0005] Boels, I.C. , Ramos, A. , Kleerebezem, M. , and de Vos, W.M. (2001) Functional analysis of the *Lactococcus lactis* galU and galE genes and their impact on sugar nucleotide and exopolysaccharide biosynthesis. Appl Environ Microbiol 67: 3033–3040. 11425718 10.1128/AEM.67.7.3033-3040.2001 PMC92977

[mbt212314-bib-0006] Boels, I.C. , Van Kranenburg, R. , Kanning, M.W. , Chong, B.F. , De Vos, W.M. , and Kleerebezem, M. (2003) Increased exopolysaccharide production in *Lactococcus lactis* due to increased levels of expression of the NIZO B40 eps gene cluster. Appl Environ Microbiol 69: 5029–5031. 12902307 10.1128/AEM.69.8.5029-5031.2003 PMC169107

[mbt212314-bib-0007] Broadbent, J.R. , McMahon, D.J. , Welker, D.L. , Oberg, C.J. , and Moineau, S. (2003) Biochemistry, genetics, and applications of exopolysaccharide production in *Streptococcus thermophilus*: a review. J Dairy Sci 86: 407–423. 12647947 10.3168/jds.S0022-0302(03)73619-4

[mbt212314-bib-0008] Cieslewicz, M.J. , Kasper, D.L. , Wang, Y. , and Wessels, M.R. (2001) Functional analysis in type Ia group B *Streptococcus* of a cluster of genes involved in extracellular polysaccharide production by diverse species of streptococci. J Biol Chem 276: 139–146. 11027683 10.1074/jbc.M005702200

[mbt212314-bib-0009] Denou, E. , Pridmore, R.D. , Berger, B. , Panoff, J.M. , Arigoni, F. , and Brussow, H. (2008) Identification of genes associated with the long‐gut‐persistence phenotype of the probiotic *Lactobacillus johnsonii* strain NCC533 using a combination of genomics and transcriptome analysis. J Bacteriol 190: 3161–3168. 18223069 10.1128/JB.01637-07 PMC2347406

[mbt212314-bib-0010] Dertli, E. , Colquhoun, I.J. , Gunning, A.P. , Bongaerts, R.J. , Le Gall, G. , Bonev, B.B. , *et al* (2013) Structure and biosynthesis of two exopolysaccharides produced by *Lactobacillus johnsonii* FI9785. J Biol Chem 288: 31938–31951. 24019531 10.1074/jbc.M113.507418 PMC3814790

[mbt212314-bib-0011] Dertli, E. , Mayer, M.J. , and Narbad, A. (2015) Impact of the exopolysaccharide layer on biofilms, adhesion and resistance to stress in *Lactobacillus johnsonii* FI9785. BMC Microbiol 15: 8. doi: 10.1186/s12866‐015‐0347‐2 25648083 10.1186/s12866-015-0347-2 PMC4326364

[mbt212314-bib-0012] DuBois, M. , Gilles, K.A. , Hamilton, J.K. , Rebers, P.A. , and Smith, F. (1956) Colorimetric method for determination of sugars and related substances. Anal Chem 28: 350–356.

[mbt212314-bib-0013] Fanning, S. , Hall, L.J. , Cronin, M. , Zomer, A. , MacSharry, J. , Goulding, D. , *et al* (2012) Bifidobacterial surface‐exopolysaccharide facilitates commensal‐host interaction through immune modulation and pathogen protection. Proc Natl Acad Sci USA 109: 2108–2113. 22308390 10.1073/pnas.1115621109 PMC3277520

[mbt212314-bib-0014] Horn, N. , Wegmann, U. , Narbad, A. , and Gasson, M.J. (2005) Characterisation of a novel plasmid p9785S from *Lactobacillus johnsonii* FI9785. Plasmid 54: 176–183. 16122563 10.1016/j.plasmid.2005.01.005

[mbt212314-bib-0015] Horn, N. , Wegmann, U. , Dertli, E. , Mulholland, F. , Collins, S.R.A. , Waldron, K.W. , *et al* (2013) Spontaneous mutation reveals influence of exopolysaccharide on *Lactobacillus johnsonii* surface characteristics. PloS ONE 8: e59957. 23544114 10.1371/journal.pone.0059957 PMC3609815

[mbt212314-bib-0016] Huang, L. , Takahashi, R. , Kobayashi, S. , Kawase, T. , and Nishinari, K. (2002) Gelation behavior of native and acetylated konjac glucomannan. Biomacromolecules 3: 1296–1303. 12425668 10.1021/bm0255995

[mbt212314-bib-0017] Jolly, L. , and Stingele, F. (2001) Molecular organization and functionality of exopolysaccharide gene clusters in lactic acid bacteria. Int Dairy J 11: 733–745.

[mbt212314-bib-0018] Kajimura, J. , Rahman, A. , Hsu, J. , Evans, M.R. , Gardner, K.H. , and Rick, P.D. (2006) O acetylation of the enterobacterial common antigen polysaccharide is catalyzed by the product of the *yiaH* gene of *Escherichia coli* K‐12. J Bacteriol 188: 7542–7550. 16936038 10.1128/JB.00783-06 PMC1636290

[mbt212314-bib-0019] Kitazawa, H. , Harata, T. , Uemura, J. , Saito, T. , Kaneko, T. , and Itoh, T. (1998) Phosphate group requirement for mitogenic activation of lymphocytes by an extracellular phosphopolysaccharide from *Lactobacillus delbrueckii* ssp. *bulgaricus* . Int J Food Microbiol 40: 169–175. 9620124 10.1016/s0168-1605(98)00030-0

[mbt212314-bib-0020] La Ragione, R.M. , Narbad, A. , Gasson, M.J. , and Woodward, M.J. (2004) *In vivo* characterization of *Lactobacillus johnsonii* FI9785 for use as a defined competitive exclusion agent against bacterial pathogens in poultry. Lett Appl Microbiol 38: 197–205. 14962040 10.1111/j.1472-765x.2004.01474.x

[mbt212314-bib-0021] Lebeer, S. , Vanderleyden, J. , and De Keersmaecker, S.C. (2008) Genes and molecules of lactobacilli supporting probiotic action. Microbiol Mol Biol Rev 72: 728–764. 19052326 10.1128/MMBR.00017-08 PMC2593565

[mbt212314-bib-0022] Lebeer, S. , Verhoeven, T.L. , Francius, G. , Schoofs, G. , Lambrichts, I. , Dufrene, Y. , *et al* (2009) Identification of a gene cluster for the biosynthesis of a long, galactose‐rich exopolysaccharide in *Lactobacillus rhamnosus* GG and functional analysis of the priming glycosyltransferase. Appl Environ Microbiol 75: 3554–3563. 19346339 10.1128/AEM.02919-08 PMC2687306

[mbt212314-bib-0023] Levander, F. , Svensson, M. , and Radstrom, P. (2002) Enhanced exopolysaccharide production by metabolic engineering of *Streptococcus thermophilus* . Appl Environ Microbiol 68: 784–790. 11823219 10.1128/AEM.68.2.784-790.2002 PMC126717

[mbt212314-bib-0024] Looijesteijn, P.J. , Boels, I.C. , Kleerebezem, M. , and Hugenholtz, J. (1999) Regulation of exopolysaccharide production by *Lactococcus lactis* subsp. *cremoris* by the sugar source. Appl Environ Microbiol 65: 5003–5008. 10543815 10.1128/aem.65.11.5003-5008.1999 PMC91673

[mbt212314-bib-0025] Maguin, E. , Prevost, H. , Ehrlich, S.D. , and Gruss, A. (1996) Efficient insertional mutagenesis in lactococci and other gram‐positive bacteria. J Bacteriol 178: 931–935. 8550537 10.1128/jb.178.3.931-935.1996 PMC177749

[mbt212314-bib-0026] Mozzi, F. , Gerbino, E. , Font de Valdez, G. , and Torino, M.I. (2009) Functionality of exopolysaccharides produced by lactic acid bacteria in an *in vitro* gastric system. J Appl Microbiol 107: 56–64. 19291238 10.1111/j.1365-2672.2009.04182.x

[mbt212314-bib-0027] Nakajima, H. , Suzuki, Y. , and Hirota, T. (1992) Cholesterol lowering activity of ropy fermented milk. J Food Sci 57: 1327–1329.

[mbt212314-bib-0028] Peant, B. , LaPointe, G. , Gilbert, C. , Atlan, D. , Ward, P. , and Roy, D. (2005) Comparative analysis of the exopolysaccharide biosynthesis gene clusters from four strains of *Lactobacillus rhamnosus* . Microbiol 151: 1839–1851. 10.1099/mic.0.27852-0 15941992

[mbt212314-bib-0029] Reid, G. , and Burton, J. (2002) Use of *Lactobacillus* to prevent infection by pathogenic bacteria. Microbes Infect 4: 319–324. 11909742 10.1016/s1286-4579(02)01544-7

[mbt212314-bib-0030] Riley, L.M. , Weadge, J.T. , Baker, P. , Robinson, H. , Codee, J.D. , Tipton, P.A. , *et al* (2013) Structural and functional characterization of *Pseudomonas aeruginosa* AlgX: role of AlgX in alginate acetylation. J Biol Chem 288: 22299–22314. 23779107 10.1074/jbc.M113.484931 PMC3829321

[mbt212314-bib-0031] Robijn, G.W. , van den Berg, D.J. , Haas, H. , Kamerling, J.P. , and Vliegenthart, J.F. (1995) Determination of the structure of the exopolysaccharide produced by *Lactobacillus sake* 0‐1. Carbohydr Res 276: 117–136. 8536250 10.1016/0008-6215(95)00172-p

[mbt212314-bib-0032] Sims, I.M. , Frese, S.A. , Walter, J. , Loach, D. , Wilson, M. , Appleyard, K. , *et al* (2011) Structure and functions of exopolysaccharide produced by gut commensal *Lactobacillus reuteri* 100‐23. ISME J 5: 1115–1124. 21248858 10.1038/ismej.2010.201 PMC3146279

[mbt212314-bib-0033] Stingele, F. , Newell, J.W. , and Neeser, J.R. (1999) Unraveling the function of glycosyltransferases in *Streptococcus thermophilus* Sfi6. J Bacteriol 181: 6354–6360. 10515925 10.1128/jb.181.20.6354-6360.1999 PMC103770

[mbt212314-bib-0034] Tallon, R. , Bressollier, P. , and Urdaci, M.C. (2003) Isolation and characterization of two exopolysaccharides produced by *Lactobacillus plantarum* EP56. Res Microbiol 154: 705–712. 14643409 10.1016/j.resmic.2003.09.006

[mbt212314-bib-0035] Vu, B. , Chen, M. , Crawford, R.J. , and Ivanova, E.P. (2009) Bacterial extracellular polysaccharides involved in biofilm formation. Molecules 14: 2535–2554. 19633622 10.3390/molecules14072535 PMC6254922

[mbt212314-bib-0036] Walter, J. , Schwab, C. , Loach, D.M. , Ganzle, M.G. , and Tannock, G.W. (2008) Glucosyltransferase A (GtfA) and inulosucrase (Inu) of *Lactobacillus reuteri* TMW1.106 contribute to cell aggregation, *in vitro* biofilm formation, and colonization of the mouse gastrointestinal tract. Microbiol 154: 72–80. 10.1099/mic.0.2007/010637-0 18174127

[mbt212314-bib-0037] Wegmann, U. , Overweg, K. , Horn, N. , Goesmann, A. , Narbad, A. , Gasson, M.J. , and Shearman, C. (2009) Complete genome sequence of *Lactobacillus johnsonii* FI9785, a competitive exclusion agent against pathogens in poultry. J Bacteriol 191: 7142–7143. 19767436 10.1128/JB.01182-09 PMC2772503

[mbt212314-bib-0038] Welman, A.D. , and Maddox, I.S. (2003) Exopolysaccharides from lactic acid bacteria: perspectives and challenges. Trends Biotechnol 21: 269–274. 12788547 10.1016/S0167-7799(03)00107-0

