# Supplementary material for: EpsA is an essential gene in exopolysaccharide production in L actobacillus johnsonii FI9785
Source: Microb Biotechnol. 2015 Sep 24;9(4):496–501. doi: 10.1111/1751-7915.12314 (PMC4919991; doi:10.1111/1751-7915.12314)
Supplement: Supplementary file 1 — Fig. S1. 600 MHz 1H NMR spectra of EPS (300°K, D2O) isolated from WT, ΔepsA and ΔepsA::pepsA strains. Table S1. Primers designed for epsA, 16S and gyrB genes for qPCR analysis. [file MBT2-9-496-s001.docx]

**SUPPORTING INFORMATION**

**1. NMR spectra of EPSs isolated from wild type, knockout and complemented strains**

**
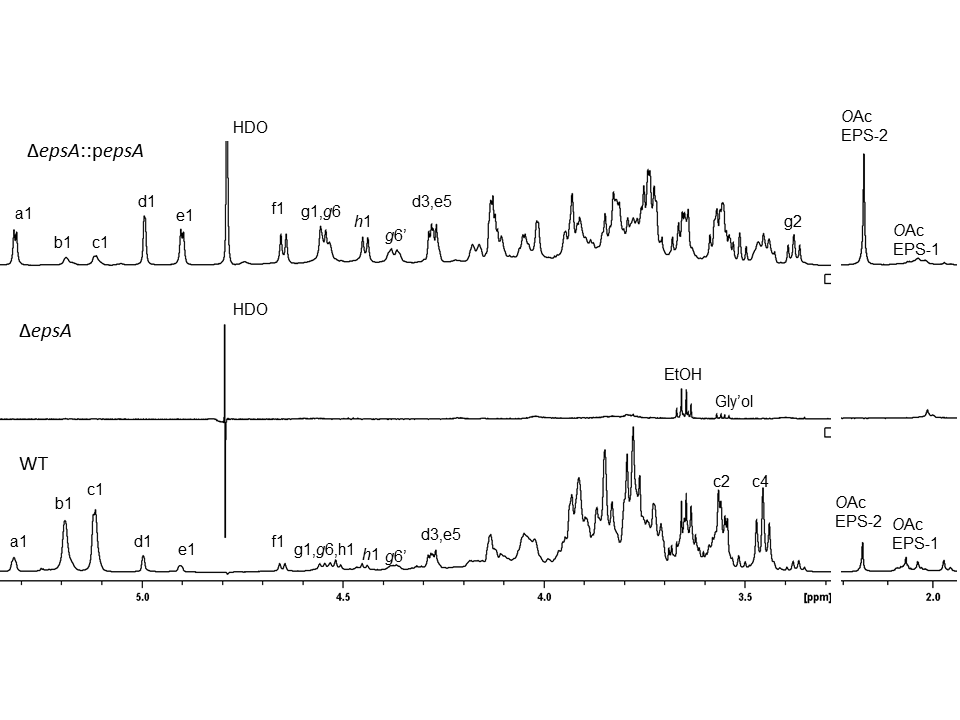
**

**Figure S1.** 600MHz ^1^H NMR spectra of EPS (300^0^K, D_2_O) isolated from WT, Δ*epsA* and Δ*epsA*::p*epsA* strains. The vertical gain of the *O*Ac region (2.0-2.2 ppm) has been reduced x2 relative to the main carbohydrate signal region (3.3-5.4 ppm). The same isolation procedure was applied to obtain all three samples. For Δ*epsA* no EPS carbohydrate signals were detected: the only signals present in the carbohydrate region were from ethanol and glycerol which arose from the isolation procedure. Selected signals from the WT and Δ*epsA*::p*epsA* strains have been labelled as in Figure 2 in the main text i.e. the H1 signal from unit a is labelled a1 (also see Figure 2 for the structures of EPS-1 and EPS-2 and the coding of their sugar residues).

It is clear from Figure S1 that EPS-1 (units b and c) is the major component of WT and EPS-2 (units a, d, e, f, g, h) is the major component of Δ*epsA*::p*epsA*. The fine structure of carbohydrate signals in the anomeric region (~4.2-5.4 ppm) is affected by the presence and level of *O*Ac substituents in the EPS. It was explained previously (Dertli et al., 2013) that the *O*Ac singlet at 2.15 ppm was associated with EPS-2 since the loss of this acetyl substituent was accompanied by changes to some of the EPS-2 signals in the anomeric region. However in the samples examined prior to this study EPS-2 was only partly acetylated. In Δ*epsA*::p*epsA* there is exactly one *O*Ac substituent per hexasaccharide repeating unit (integrated intensity of the 2.15 ppm (CH_3_) singlet was 3:1 relative to any of the H1 signals). This simplified the assignment of all the signals of the hexasaccharide unit (shown in Table 1), using the 2D NMR procedures previously described in detail (Dertli et al., 2013). In particular it revealed a marked downfield displacement of the ^1^H and ^13^C shifts of g6 in the acetylated EPS-2 hexasaccharide unit compared with the corresponding deacetylated unit (Table 4 in Dertli et al., 2013) coupled with an upfield ^13^C shift of the neighbouring g5. As explained in the text this is consistent with the location of the acetyl substituent on *O*6 of residue g. There are also secondary spatial proximity effects on the ^1^H shifts of h1 and a1 in the neighbouring residues to g. Some of these affected peaks in the acetylated unit have been labelled in italic in Figure S1: *g*6 and *g*6´, *h*1. The WT sample represents an example of partially acetylated EPS-2 (ratio of *O*Ac: a1 = 1.5): signals h1 and *h*1 can both be seen and arise from non-acetylated and acetylated hexasaccharide units respectively. In this sample only 50% of EPS-2 g residues are acetylated at *O*6.

**2. Deletion, complementation and expression analysis of *epsA***

**Deletion of the *epsA* gene from the *eps* cluster of *L. johnsonii* FI9785**

The *epsA* gene was deleted from the *L. johnsonii* FI9785 chromosome using the thermosensitive vector pG^+^host9 (Maguin *et al.*, 1996). Firstly, 390 bp of the *epsA* gene and some upstream sequence were amplified from genomic DNA using primers 5epsA_KpnF (5’-AAAGGTACCAAATTAAATAACAAGAG-3’) (altered nucleotides underlined throughout) and epsA_R1 (5’-CGGTAAGTTAACTTTCATATCTCG-3’). The partial *epsA* product was restricted using KpnI and XhoI and subcloned into restricted pG^+^host9. The ligation product was transformed into electrocompetent *E. coli* MC1022 and positive colonies were selected with erythromycin (400 μg/ml). To produce the *epsA* knockout cassette, 539 bp from the 5’ untranslated region of the *epsB* gene was amplified and XhoI / HindIII sites were generated using primers 5epsB_XhoF (GACTCGAGAATAGGAAAAAGTGG) and epsB_HindIIIR (GCAAAAGCTTGTGACTTTTCTG). The partial *epsB* product was then restricted and subcloned into pG^+^host9::p*epsA*. The deletion plasmid pG^+^host9::p*epsA_B* was transformed into *L. johnsonii* FI9785 by electroporation (Horn *et al.*, 2005) and single and double crossovers were induced as described by Maguin *et al*., (Maguin *et al.*, 1996) using 30°C as the permissive temperature and 42°C as the non-permissive temperature used to induce integration, giving a final product with a deletion of 630 bp from the *epsA* gene (*L. johnsonii* Δ*epsA*, FI10785).

***epsA* complementation**

For complementation, the *epsA* gene was PCR amplified from FI9785 genomic DNA using the primer pair 5epsA_NcoI (5’-ATACCATGGATCATAAGAATAGTG-3’) and epsANcoI_R (5’-TTTCCATGGTTTCCTATTCTCC-3’) to produce a 1023 bp product with NcoI sites. This product was then digested and ligated into the expression vector pFI2560 (Horn *et al.*, 2013). The construct (pFI2563) was transformed into *L. johnsonii* *ΔepsA* to produce *L. johnsonii* Δ*epsA*::p*epsA* (FI10763)

**Analysis of *epsA* gene expression levels by quantitative real-time PCR (qPCR)**

For qPCR analysis, total RNA was extracted from 3 ml of mid- to late exponential phase cultures of *L. johnsonii* FI9785 wild type, *∆epsA* and ∆*epsA*::p*epsA* strains grown in MRS with glucose. The RNA was stabilized prior to extraction with RNAprotect Bacteria Reagent (Qiagen) then extracted after an enzymatic lysis followed by a mechanical disruption of the cells using the RNeasy Mini Kit (Qiagen). Genomic DNA contamination was removed by DNAse treatment using TURBO DNA-free kit (Life Technologies). Expression of the *epsA* gene in these three strains was quantified by qPCR on an Applied Biosystems 7500 Real-Time PCR system (Life Technologies) using the 16S and *gyrB* genes as housekeeping genes. Pairs of primers were designed for *epsA*, 16S and *gyrB* genes using ProbeFinder version 2.45 (Roche) to give amplicons of 60-80 bp (Supplementary Table 1). Calibration curves were prepared in triplicate for each pair of primers using 2.5-fold serial dilutions of *L. johnsonii* FI9785 genomic DNA. The standard curves showed a linear relationship of log input DNA (ng) vs. the threshold cycle (C_T_), with acceptable values for the slopes and the regression coefficients (R^2^). The dissociation curves were also performed to check the specificity of the amplicons.

DNAse-treated RNA (1 µg) was converted into cDNA using the QuantiTect® Reverse Transcription kit (Qiagen). Each 10 µl-qPCR reaction was then carried out in triplicate with 1 µl of a 20-fold diluted sample and 0.2 µM of each primer, using the QuantiFast SYBR Green PCR kit (Qiagen) and conditions of 95°C for 5 min followed by 35 cycles of 95°C for 10 s and 60°C for 30 s. All sample and primer combinations were assessed in triplicate. Control PCR conﬁrmed that there was no background contamination or residual chromosomal DNA. PCR speciﬁcity and product detection was checked post ampliﬁcation by examining the temperature-dependent melting curves of the PCR products. Generation of quantitative data by real-time PCR is based on the number of cycles needed for amplification generated fluorescence to reach a specific threshold of detection (the C_T_ value) for each strain. For relative quantification of *epsA* gene expression in each strain, the *epsA* gene expressions were compared with the housekeeping gene *gyrB* using the 2^-∆∆CT^ methodology.

**Supplementary Table 1.** Primers designed for *epsA*, 16S and *gyrB* genes for qPCR analysis.

**Primer Sequence (5’-3’) Length (nt) T_m_** °C **Amplicon**

**epsA_1R** Tcttgatcgttttaacagtttcatct 26 59

**epsA_1F** Ccagctaagattaatgcagccta 23 59 64 nt

**epsA_2R** Tcactaatttcattactcatcggatt 26 59

**epsA_2F** Ggttattatcgcttggcacaat 22 60 75 nt

**16S_R** Ccgaactgagaacggcttta 20 60

**16S_F** Ggtacaacgagaagcgaacc 20 59 61 nt

**gyrB_R** Cttgaagaacatggaacaatcg 22 59

**gyrB_F** Cgtcgaaagttgtagtttcggta 23 60 74 nt
